# RETRACTION: Wound Complications Frequency in Heart Transplant Recipients on Mammalian Target of Rapamycin Inhibitors: A Meta‐Analysis

**DOI:** 10.1111/iwj.70208

**Published:** 2025-02-02

## Abstract

**RETRACTION:** ZhuS. , YuW. , GaoJ. , and XiongJ. , “Wound Complications Frequency in Heart Transplant Recipients on Mammalian Target of Rapamycin Inhibitors: A Meta‐Analysis,” International Wound Journal 20, no. 9 (2023): 3491–3497, 10.1111/iwj.14221.37165731 PMC10588318

The above article, published online on 10 May 2023, in Wiley Online Library (http://onlinelibrary.wiley.com/), has been retracted by agreement between the journal Editor in Chief, Professor Keith Harding; and John Wiley & Sons Ltd. Following an investigation by the publisher, all parties have concluded that this article was accepted solely on the basis of a compromised peer review process. The editors have therefore decided to retract the article. The authors did not respond to our notice regarding the retraction.

**RETRACTION:** S. Zhu , W. Yu , J. Gao , and J. Xiong , “Wound Complications Frequency in Heart Transplant Recipients on Mammalian Target of Rapamycin Inhibitors: A Meta‐Analysis,” International Wound Journal 20, no. 9 (2023): 3491–3497, 10.1111/iwj.14221.37165731 PMC10588318

The above article, published online on 10 May 2023, in Wiley Online Library (http://onlinelibrary.wiley.com/), has been retracted by agreement between the journal Editor in Chief, Professor Keith Harding; and John Wiley & Sons Ltd. Following an investigation by the publisher, all parties have concluded that this article was accepted solely on the basis of a compromised peer review process. The editors have therefore decided to retract the article. The authors did not respond to our notice regarding the retraction.

